# Non-Linear Dynamical Analysis of Resting Tremor for Demand-Driven Deep Brain Stimulation

**DOI:** 10.3390/s19112507

**Published:** 2019-05-31

**Authors:** Carmen Camara, Narayan P. Subramaniyam, Kevin Warwick, Lauri Parkkonen, Tipu Aziz, Ernesto Pereda

**Affiliations:** 1 Department of Computer Science, Carlos III University of Madrid, 28903 Madrid, Spain; 2 Centre for Biomedical Technology, Technical University of Madrid, 28040 Madrid, Spain; eperdepa@ull.edu.es; 3 Department of Neuroscience and Biomedical Engineering, School of Science, Aalto University, FI-00076 Helsinki, Finland; narayan.subramaniyam@aalto.fi (N.P.S.); lauri.parkkonen@aalto.fi (L.P.); 4 Vice Chancellors Office, Coventry University, Coventry CV1 5FB, UK; k.warwick@reading.ac.uk; 5 Nuffield Department of Clinical Neuroscience, University of Oxford, Oxford OX1 2JD, UK; tipu.aziz@nds.ox.ac.uk; 6 Department of Industrial Engineering, Laboratory of Electrical Engineering and Bioengineering, Universidad de La Laguna, 38200 Tenerife, Spain

**Keywords:** nonlinear dynamics, Recurrence Networks (RNs), Support Vector Machine (SVM), Deep Brain Stimulation (DBS), Parkinson’s Disease (PD), Local Field Potentials (LFPs)

## Abstract

Parkinson’s Disease (PD) is currently the second most common neurodegenerative disease. One of the most characteristic symptoms of PD is resting tremor. Local Field Potentials (LFPs) have been widely studied to investigate deviations from the typical patterns of healthy brain activity. However, the inherent dynamics of the Sub-Thalamic Nucleus (STN) LFPs and their spatiotemporal dynamics have not been well characterized. In this work, we study the non-linear dynamical behaviour of STN-LFPs of Parkinsonian patients using ε-recurrence networks. RNs are a non-linear analysis tool that encodes the geometric information of the underlying system, which can be characterised (for example, using graph theoretical measures) to extract information on the geometric properties of the attractor. Results show that the activity of the STN becomes more non-linear during the tremor episodes and that ε-recurrence network analysis is a suitable method to distinguish the transitions between movement conditions, anticipating the onset of the tremor, with the potential for application in a demand-driven deep brain stimulation system.

## 1. Introduction

It is estimated that the number of Parkinson’s patients will outnumber those with Alzheimer’s disease by 2040, thus reaching pandemic proportions [1]. Although Parkinson’s disease is currently the second most frequent neurodegenerative disorder, in 95% of the cases, it remains an idiopathic disease [2]. Thus, there is a need for the medical and research community to understand its origin and to explore improved diagnostic methods and treatments that work better in the long term.

Parkinson’s symptomatology is diverse. Patients can experience tremor of the extremities at rest, the so-called *Resting Tremor* (RT), muscle rigidity, slow motion (bradykinesia) or difficulty in carrying out precise movements (akinesia). The patients in this study were diagnosed with *benign tremulous Parkinsonism*. This type of Parkinson’s is manifested by the following characteristics: (1) a noticeable RT; (2) symptoms not related to tremor have a confined incidence; (3) predominant lack of gait disorder; and (4) lack of impairments apart from tremor [3].

Associated with this symptomatology, clinical states can be differentiated: the Non-Tremorous resting state (*NT*), in which the patient does not experience tremor, and the Tremor state (*T*), in which the patient suffers from tremor at rest. Among them, we can find a state called Tremor Onset (*TO*), which is supposed to hold the key to understanding the transition between *NT* and *T*. A similar dynamical behaviour of the brain, transitioning between different states, can be found in other diseases. Such conditions are known as dynamical diseases [4]. As a general definition, a dynamic disease causes abnormal dynamics in a physiological control system operating within a range of control parameters [5]. Methods derived from non-linear dynamics analysis have been shown to be good at detecting key features of the different dynamical states, providing a formal understanding of how the emergence of the manifestations of dynamical diseases takes place [4,6].

An oral treatment, generally with dopaminergic effects, is usually the first option. Unfortunately, these patients have a high resistance to levodopa, even at doses above the tolerable levels [3]. Furthermore, after a few years of use, the patients begin to suffer from the so-called ON-OFF effect: ON periods, in which the drug works, along with alternate OFF periods, in which despite the patient taking the drug as prescribed, it does not perform well and the patient continues suffering from the symptoms [7]. Furthermore, the use of levodopa leads to dyskinesias (LID), causing involuntary movements that are often worse than the original PD’s symptomatology [8,9].

Deep Brain Stimulation (DBS) constitutes an alternative line of treatment in these cases. DBS consists of the surgical implantation of a neurostimulator. A neurostimulator is an Implantable Medical Device (IMD) made up of a pulse generator (IPG), and a set of electrodes, which provides High-Frequency Stimulation (HFS) to the target area, usually the Sub-Thalamic Nucleus (STN). Current neurostimulators work uninterruptedly after its implantation. As a consequence, several adverse effects have been reported in the literature, including paresthesia, cognitive or psychiatric dysfunction and even an increased risk of suicide [10,11]. Demand-driven deep brain stimulation strategies constitute an improved version of the current procedure, triggering stimulation only when necessary, which is believed to reduce the side effects [12,13,14,15]. Moreover, a closed-loop approach would extend the battery lifetime, as a result of the more efficient use of it.

The implementation of a demand-driven system requires an understanding of what features of the basal ganglia activity, the STN in the case of this study, change shortly before the symptoms appear. Understanding the tremor nature is paramount to decipher the behaviour of the STN nucleus in the different clinical states (*NT*, *TO* and *T*). However, this is challenging since STN-Local Field Potential (LFP) recordings in humans are generally acquired in 2–3 days after the surgical procedure, before the connection of the electrodes to the IPG, not being accessible any longer. Note that this procedure is carried out for research purposes. Otherwise, the electrodes are usually implanted during the surgery. Furthermore, we need to gather the neural activity when the patient (without medication and spontaneously) makes the transition from the *NT* to the *T* state. These two limitations make this type of recording difficult to obtain. In this work, we analyse four of them from our dataset, which fulfil these properties. The length of the four files studied ranged from 40 s–3.6 min (40 s, 2.13 min and 3.6 min). The objective is to characterise the dynamics of the different clinical states, with a particular interest in finding a fingerprint for the *TO* state, as this is the first step in preventing or suppressing the tremor with demand-driven DBS.

Local Field Potentials (LFPs) have been widely studied. While some researchers have explored the spectral power of the neural oscillations, usually assuming the existence of pathology when its range deviates from those present in control subjects [16,17], others have studied the connectivity between the neurons within the STN [10,18,19]. However, the inherent dynamics and spatiotemporal profile of STN-LFPs have not been characterised for their application to a demand-driven DBS system. In the cases of dynamical diseases, algorithms from the non-linear dynamical analysis are particularly attractive to find biomarkers that differ between normal and pathological states of the disease [20].

In this work, we apply time-series analysis methods derived from non-linear dynamics to the study of the STN activity under the different tremor states. A moving window Recurrence Network (RN) analysis is carried out to capture such rich dynamical behaviour. RNs are constructed based on the recurrences in the phase space. By applying network analysis methods, the dynamically-relevant structures of the time series data can be studied, extracting the geometrical properties of the attractor [21]. The study of the topological structure of the RN allows us to infer the complexity of the dynamics associated with the STN-LFP time series.

Methods based on RN constitute an excellent approach to analyse the structural complexity of neural signals, as they can be applied to short and non-stationary data, since the network properties, such as global clustering coefficient, transitivity or assortativity, can still be reliably estimated. This makes RNs an ideal method for capturing the dynamical transitions in neural data. Previous works have demonstrated the applicability of this method in detecting the onset of epileptic seizures [6,22].

The present study aims to answer two main questions: (1) Do the dynamics of STN-LFPs have a permanent character or do they change depending on the movement state that the patient is in? (2) Would it be possible to characterise some feature in the neural signal, which can aid in predicting the onset of the tremor?

## 2. Data Preparation

### 2.1. Dataset

The data consist of LFPs recording from the STN of four Parkinsonian patients. All patients were implanted with a neurostimulator at the John Radcliffe Hospital in Oxford, U.K. The proper positioning of the DBS electrodes within the STN was verified by postoperative magnetic resonance imaging.

LFPs captured the electrical activity of the neuron population in the electrode neighbourhood. A Medtronic DBS Lead Model 3387 with four electrodes (1.5 mm apart) was the device employed for the signal acquisition. Recordings were made within 2–3 days following the surgical procedure, the time in which the electrodes have not been connected to the IPG and internalized, and therefore, the signal was accessible for recording. Note that the recordings have been obtained under the DBS-OFF condition. In this regard, the validity of the proposed system in recognizing the patient’s clinical states in a DBS-ON context could be questioned. As will be explained later in Section 8.3, this will not be a problem in the new generation of neurostimulation devices, in which closed-loop strategies are framed.

Additionally, simultaneous to the LFP recording, the Electromyography (EMG) signal was acquired in order to label the data into tremorous and atremorous sections. The EMG records were taken from the extensor with the arm contralateral to the neurostimulator implantation side.

All patients gave their informed consent for inclusion before they participated in the study. The study was conducted following the Declaration of Helsinki, and the protocol was approved by the local research ethics committee of the Oxfordshire Health Authority (RECReference Number 08/H0604158).

### 2.2. Signal Preprocessing

The sampling rate of the recordings varied between 250 and 1000 Hz. In order to make the results comparable, we started by resampling the recordings so that we could apply the same processing procedure to all the data. To avoid redundant information and according to the Nyquist theorem, downsampling to 125 Hz was performed. After that, STN-LFP recordings were filtered with a 500th order [2–45]-Hz band-pass FIR filter designed with a Hamming window in which we used two seconds of real data as padding. This step eliminated the movement artefacts (around 1 Hz) and the line noise (50 Hz in Europe). Finally, the filtered data were segmented into windows of two seconds, using overlapping of 90%. Using this level of overlapping, we can seize with a high temporal resolution the dynamics of the signal.

The EMG signal during tremor is composed of bursts having frequency peaks at 30 Hz and above. The data were filtered using a two-pass procedure with a high-pass filter above 30 Hz of order 500 and designed using a Hamming window. Then, the signal was rectified using the Hilbert envelope. Finally, the rectified signal was filtered with an FIR 2–45 Hz filter, getting the EMG signal at low frequencies.

### 2.3. Data Labelling

The *NT* and *T* sections were determined by a clinical specialist in movement disorders and subsequently confirmed by the EMG signal. An additional process has been carried out for the labelling of the tremor onset *TO*. Like in previous works [19,23], the *TO* sections were identified relying on the amplitude of the filtered and rectified EMG signal. The magnitude of the EMG signal was compared against a threshold of three times the mean of the EMG amplitude in the first 5 s of the recording (which contained atremorous data). If a peak of high tremor frequency activity was detected at any point in time, the average of the following 5 s (enough to cover any period of tremor-onset) of data was calculated to confirm the tremor-onset detection. The EMG value could have been compared against a simple threshold, but in that case, the presence of small magnitude spikes might have triggered an incorrect detection.

## 3. Recurrence Networks

A dynamical system is a model in which the current state depends on the previous states and the transitional laws between them followed by the system. The state is defined through the values taken by the system variables. By connecting the different states through which the system passes, the trajectory of the system is obtained, which can be represented in the well-known phase space of the system [24].

The phase space trajectory of a dynamical system can be reconstructed from a scalar time series, by taking *m* time lagged observations of it. This is the well-known Taken’s embedding theorem [25]:

(1) x(n)=(u(n),u(n+τ),…,u(n+(m−1)τ))

where x(n)∈R, τ is the embedding delay and *m* is the embedding dimension. Taken’s theorem does not specify the values that τ and *m* should take. Here, we make use of two widely-used methods to set these parameters:

### 3.1. Embedding Delay: τ

The selection of τ has to be performed carefully, to avoid redundancy in the consecutive variables of the delayed vector. When τ is too small, no new information is extracted between successive observations, while if τ is too high, continuous observations are disconnected. To find an optimal value of τ, we used auto-mutual information. Given a scalar time series, $x_{n}$ with *n* samples, one can define the auto-mutual information function as follows [26]:

(2) $$MI\left(\tau \right)=\sum_{n=0}^{N-1}p\left(x_{n},x_{n+\tau }\right)\log\frac{p(x_{n},x_{n+\tau })}{p\left(x_{n}\right)p\left(x_{n+\tau }\right)}$$

where p(·) stands for probability. The optimal value for the embedding delay is the value of τ at which MI(τ) reaches the first local minimum [27].

### 3.2. Embedding Dimension: *m*

**Figure 1:**
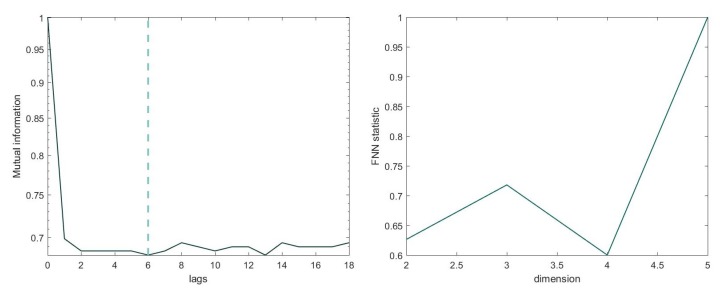
Optimal value of the parameters for an exemplary window. Left: optimal delay τ calculated with auto-mutual information. The dashed line determines the first local minimum (τ=6). Right: the minimum embedding *m* employing the False Nearest Neighbourhood (FNN) method. At m=4, the FNN statistic is zero.

For the calculation of the optimal embedding dimension, we used the False Nearest Neighbourhood (FNN) method [28], which consists of calculating the number of points along the trajectory that are neighbours for different values of *m*. The value of *m* at which the percentage of false neighbours becomes zero (or arbitrarily small, due to the effect of noise) is considered the optimal value of *m*. In order to construct RNs of the same size, the same values of τ and *m* are set across windows of each file, allowing us to compare them. Figure 1 shows the auto-mutual information and the minimum embedding *m* using the FNN method for an exemplary channel.

### 3.3. ε-Recurrence Network

From the state space, it is possible to build a complex network, on which graph-theoretical measures can be computed. In this study, a ε-RN was reconstructed. These kinds of networks are a subtype of proximity networks, in which the vertices are represented by state vectors and the edges between the vertices are defined based on the mutual closeness between different state vectors in the phase space [21,29]. We can find different proximity networks, depending on how the concept of mutual closeness is defined. In the case of a ε-RN, a fixed phase space distance ε-centred around a vertex *i* (a state vector in phase space) is defined [30]. All the vertices that fall within this volume are connected to the vertex *i* by an edge. Such a network is both undirected and symmetric.

ε-RNs are based on the recurrences in phase space and are obtained by reinterpreting the recurrence matrix as the adjacency matrix of a complex network [21,30]. A recurrence matrix represents the distance between the pairs of state vectors. It can be defined as [31]:

(3) $$R_{i,j}=\Theta (\varepsilon -\|x_{i}-x_{j}\left\|\right)$$

where ε is the recurrence threshold, Θ(·) is the Heaviside function and ‖·‖ is a distance norm. Here, we used the maximum norm as a distance norm. Instead of fixing ε, we fixed the recurrence rate RR=0.03, so that we obtained RNs with a similar number of edges across windows, which made it possible to compare them. This threshold determines the maximum spatial distance of neighbouring states.

The recurrence matrix is binary and symmetric. Each vertex *i* represents a state vector $x_{i}$. The entry is one if the distance between two states is less than the defined threshold, zero otherwise.

The adjacency matrix can be obtained from the recurrence matrix by removing the self-loops, that is subtracting the identity matrix:

(4) A=R−I

*A* represents an undirected, unweighted complex network known as a recurrence network. From it, we can characterize the dynamically-invariant properties of the neural dynamical system by using graph theoretical methods.

## 4. Network Measures

By applying network analysis methods, the dynamically-relevant structures underlying the time series can be studied by extracting the geometrical properties of the attractor. The network measures used in this study are presented in this section.

### 4.1. Global Clustering Coefficient

The local clustering coefficient for a vertex *i* represents the probability that two randomly-chosen vertices *j* and *q* are, themselves, neighbours. The local clustering coefficient of a vertex *i* can be given by:

(5) $$C_{i}=\frac{\sum_{j,q}A(i,j)A(j,q)A(q,i)}{k_{i}\left(k_{i}-1\right)}$$

where *k* is the degree of a vertex. Interpreting this coefficient in the context of RNs, $C_{i}$ is a measure of the fraction of vertices in the ε-neighbourhood of a given vertex that is itself mutually ε-close (within the same circumference). $C_{i}$ is averaged over all the vertices in the network to obtain the global clustering coefficient:

(6) $$C=\frac{1}{N}\sum_{I=1}^{N}C_{i}$$

### 4.2. Transitivity Dimension

The concept of transitivity is very similar to that of clustering. It measures the fraction of triples in the network that form triangles. The main difference is that the transitivity coefficient is normalized by the value of the whole network, a quality that makes transitivity more robust compared to clustering against the presence of nodes with a low degree. The transitivity is defined by [32] as:

(7) $$T\left(G\right)=\frac{3\delta (G)}{T(G)}$$

where T(G) and δ(G) are the total number of triples and triangles in the network, respectively. In terms of the recurrence matrix, *T* can be defined as:

(8) $$T=\frac{\sum_{i,j,q=1}^{N}A(i,j)A(j,q)A(q,i)}{\sum_{i,j,q=1}^{N}A(i,j)A(q,i)}$$

### 4.3. Assortativity

A network is assortative if the vertices with a similar degree tend to connect. The fact that a recurrence matrix is assortative means that the density of states within the ε-neighbourhood changes slowly and continuously. This coefficient is calculated by the Pearson product-moment correlation of the vertex degrees on either end of all the edges [33]:

(9) $$A=\frac{\frac{1}{N}\sum_{j>i}k_{i}k_{j}A\left(i,j\right)-{\left[\;\frac{1}{N}\sum_{j>i}\frac{1}{2}\left(k_{i}+k_{j}\right)A\left(i,j\right)\right]}^{2}}{\frac{1}{N}\sum_{j>i}\frac{1}{2}\left(k_{i}^{2}+k_{j}^{2}\right)A\left(i,j\right)-{\left[\;\frac{1}{N}\sum_{j>i}\frac{1}{2}\left(k_{i}+k_{j}\right)A\left(i,j\right)\right]}^{2}}$$

## 5. Moving Window ε-Recurrence Network Analysis

In this work, we used moving window ε-recurrence network analysis to compute the global measures C,T,A. The time series was previously divided into two-second windows with 90% overlap. In order to get the temporal profile of global network measures, we assigned the global measure to the mid-point of each window. τ and *m* have been set on the first local minimum of the auto-mutual information and FNN method, respectively, and the recurrence rate *RR* was set to 0.03.

**Figure 2:**
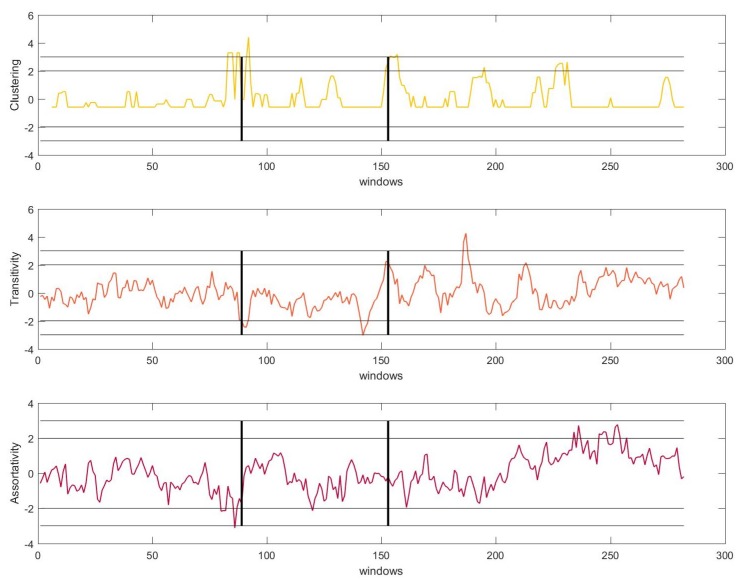
Moving window ε-recurrence network analysis showing the median moving average of clustering, transitivity and assortativity, before, during and after the start of the tremor, in that order. The left and right black vertical lines represent the transition from Non-Tremorous resting state (*NT*) to Tremor Onset (*TO*) and from *TO* to Tremor state (*T*), respectively. The horizontal lines represent the ±2 and ±3 standard deviation thresholds for statistical significance.

To determine whether it is possible to anticipate the beginning of a tremor episode, a moving median filter over the signals C,T,A was applied. This filtering allows one to smooth out short-term fluctuations and to highlight the real transients in the STN-LFP signal. The ±2 and ±3 standard deviations of the moving median filter were established to determine the statistical significance of the transients (peaks henceforth). Figure 2 shows the results of the analysis of one of the recordings.

Clustering (C): We witnessed a low level of *C* during *NT*, which increased abruptly a few seconds before the onset of the *TO* (p<0.01). The peaks described an abrupt increase in the non-linearity in the system (for simplicity, we will talk about peaks instead of an increase in the non-linearity henceforth). Thereupon, the level of *C* decreased again, although it remained higher than its level during *NT*. A few seconds before the onset of the resting tremor *T*, we again detected an increase of *C* (p<0.01). During the *T* section, significant peaks (p<0.05) were detected.

Transitivity (T): The levels of transitivity remain insignificant during *NT*. In both transition periods, we detected an increase in the level of *T* (p<0.05). During the tremor section, the amplitude of the peaks increased, surpassing in some cases the +3 standard deviation.

Assortativity (A): *A* had the same behaviour as transitivity, in that its fluctuations remained insignificant during the entire *NT* period. However, it marked the transition windows by becoming significant (p<0.05) and increasing in amplitude in *TO* and *T*.

Equivalent results were found for all files. Importantly, we found significant peaks before the transition of movement conditions for all the measures and files. These peaks were significant at the 99% and 95% levels, which implies that in all cases, the onset of the tremor can be predicted in advance by this method. Based on the obtained results, we can draw the following conclusions:

- The trends of the different measures were quite similar since all of them exhibited a shift in their dynamics near the beginning of the *TO* episode and before the *T* episode. All measures detected the tremor efficiently before its appearance and therefore before the patient showed any physical symptoms. This fact made these measures good candidates for their application in a demand-driven DBS system.
- During tremor episodes, *T* and *A* displayed a growing trend, while *C* exhibited the behaviour of shifting its dynamics more abruptly. The behaviour during *NT* and *TO* was similar across all the measures.

## 6. System

The objective of the system was to detect the tremor through the STN-LFP signal recorded by the electrodes, through the network features studied. The aim was to provide stimulation as soon as symptoms were detected, or ideally shortly before, and to stop stimulation as soon as atremorous instances were sensed. In this way, the system will be efficient concerning the treatment of symptomatology, as well as with the use of the battery.

As previously mentioned, it was within the section labelled as *T* when the patient began to experience physically appreciable tremor, as determined by clinicians with expertise in movement disorders.

From the moment a tremor window arrives until the system classifies it as such and orders the stimulation to begin, a few seconds may pass. Therefore, it would be interesting to detect within the STN signal some event that anticipates the tremor episode, i.e., the ideal solution would be to detect the tremor before the patient begins to experience it physically, using those seconds to turn on the stimulation. In other words, ideally, a demand-driven DBS system must detect the tremor when the patient is in a *TO* episode.

With this goal in mind, the dynamics of the STN signal have been studied following the methods presented in Section 5, since as we have seen, the network measures have proven to be outstanding at capturing changes in the non-linearity of the system. These changes were especially noteworthy in the *TO* state, detecting a peak that reached values of significance of p< 0.01 in all the studied recordings.

### 6.1. Start and Stop Stimulation Decision

**Figure 3:**
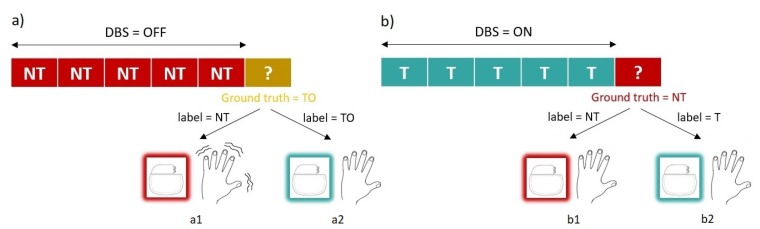
This figure represents the four cases that can take place in our system when turning on/off the stimulation. (**a**) The Implantable Medical Device (IMD) is not stimulating, and a *TO* sample arrives (the ground-truth of the sample is therefore TO). If the system fails to classify that sample, the stimulation will remain OFF, and the patient will begin to tremble after a few seconds (Scenario a1 in the figure). If on the contrary, the system correctly identifies the sample as *TO*, it will order to start the stimulation (Scenario a2 in the figure). (**b**) If while the system is stimulating, an *NT* sample arrives: (the ground-truth of the sample is therefore *NT*): If the system correctly detects this new clinical state, it will turn OFF the stimulation, as it is no longer necessary (Scenario b1 in the figure), while if the detection fails, the system will continue to stimulate (Scenario b1 in the figure). However, in this case, contrary to what happens in Scenario a1, this will have no physical effects on the patient.

To be valid for its purpose, the system must be able to recognise two main conditions: *NT* and *TO* instances. Notice that we had three classes (*NT, TO* and *T*); however, the system only needed to learn the first two to be able to carry out the two instructions that the system performed: start and stop stimulation. Notice also that the accurate detection of *TO* was more critical than the detection of *NT*. This was because if a delay existed in stopping the stimulation as a consequence of not correctly classifying an instance of *NT*, it would not have any effect on the symptomatology, going unnoticed by the patient. However, the same delay in detecting a tremor instance would lead to the patient experiencing tremor as many seconds as windows the system would need to perform a correct classification. The system has to avoid this situation, therefore, and give the accuracy shown detecting a peak of non-linearity within the *TO* state, and the system will base the decision to start stimulation on the presence of that peak. These possible scenarios are depicted in Figure 3.

However, this non-linearity peak did not occur when the patient transits from the *T* to the *NT* state, making a system based solely on this method not capable of detecting the stop condition. This would lead to a situation in which the IMD would stimulate in a continuous way (which is the current state that we were trying to improve). Therefore, to detect the stop condition, the system will made use of an SVM classifier trained per patient using ten-fold cross-validation. Support Vector Machines (SVM) are algorithms that create a non-linear discriminative classifier, determined by an optimal hyperplane that separates the instances of different classes, implicitly mapping the inputs into high-dimensional feature spaces (the well-known kernel trick) [34]. Once the system has learned the mapping function, the new and unlabelled instances will be mapped into some of the created regions, adopting the label of that region.

### 6.2. System Model

The system operated as follows: Assuming the system was running, at time *t*, a new signal window arrived at the IMD. It preprocessed the signal as described in Section 2, calculated the network measures as described in Section 4 and Section 5 and stored the results in memory. Notice that the system only maintained in memory the four windows before which it was evaluated. If DBS was OFF, the system had to decide if it would turn on the stimulation or continue just sensing the signal. For that, it averaged the current window with the four previous ones. Depending on the results: (a) If the result exceeded two standard deviations from the subject’s baseline, a non-linearity peak was detected, indicating that the patient was within the *TO* state, and therefore, the decision made by the system would be to turn on the stimulation. (b) The system would continue in standby otherwise.

**Figure 4:**
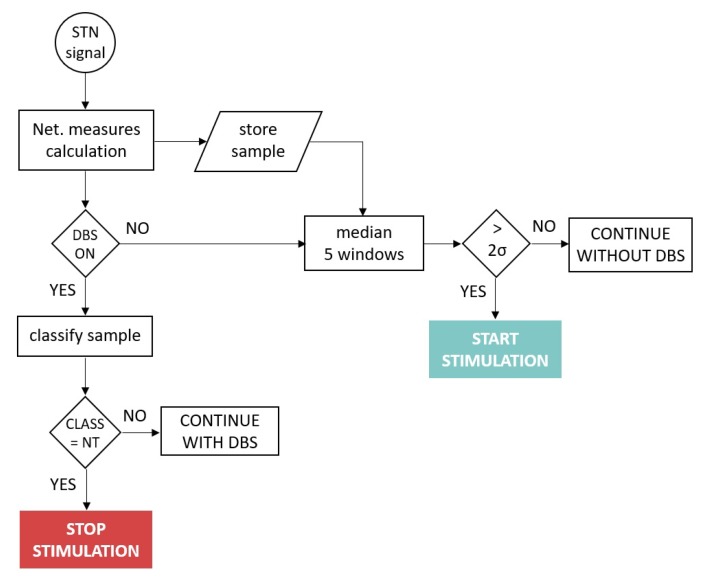
Flow diagram of the system operating mode.

Nevertheless, if in time *t*, the DBS was ON, the decision that the system must make was whether to turn off the stimulation. For this purpose, the trained SVM model would classify the sample. If it belonged to *NT*, the IMD would order to stop the stimulation. It would continue stimulating otherwise. The flow diagram of the system operating mode is depicted in Figure 4.

### 6.3. System Performance

This section discusses the validity of the proposed system. Validity reflects the accuracy of the system, and it is measured by sensitivity and specificity. Sensitivity is the proportion of true positives, and specificity is the proportion of true negatives that are correctly identified by the system. Besides, we were interested in measuring the False Positive (FPR) and False Negative Rates (FNR). FPR (α or type I error) measured the percentage of cases in which the null hypothesis was correct, but was rejected, while FNR (β or type II error) measured the percentage of cases in which the null hypothesis was false, but was accepted.

In a demand-driven DBS system, the two main actions to be taken are when to turn on and when to turn off the stimulation. The validity of the model in each of these actions is evaluated here.

**Shut down the stimulation:**

In this usage scenario, the system was stimulating (DBS ON), and it had to decide whether to stop the stimulation, i.e., the system was registering *T* samples (subthalamic signal associated with the tremor regarding the extracted features), but at a certain point, began to register *NT* samples (subthalamic signal associated with the atremorous state regarding the extracted features).

Sensitivity here is the ability of the system to classify a sample as *T* correctly, while specificity is the ability to classify a sample as *NT* correctly. A false positive in this scenario represents that the system classified an arriving sample as *NT* being *T*. While a false negative represents that an arriving sample was classified as *T* being *NT*.

The SVM module was trained to discriminate these two types of samples. Its performance is presented in Table 1. In this scenario, we wanted the system to have a high degree of specificity and a low percentage of FPR.

In the case of Patient 2, there was a specificity of 100% and an FPR of zero. This is the ideal case. One hundred percent of the *NT* samples were identified without failure. Nevertheless, in the case of Patient 3, there was a 90.8 specificity and an FPR of 9.2. This means that about nine out of 10 *NT* samples were evaluated correctly, but one out of 10 were incorrectly classified as tremor.

Notice the implication that an FPR ≠ 0 has on this scenario: If the window being evaluated was incorrectly classified as tremorous, the system would continue stimulating, and it would evaluate the next window. Continued stimulating had no effect on the symptomatology and went unnoticed for the patient (as represented in Figure 3b2). We were interested in having high specificity, but the fact of not reaching 100% was not critical.

**Start up the stimulation:**

Contrary to the previous use case, in this usage scenario, the system was in standby, sensing. For each incoming window, the system must decide whether to start stimulation, i.e, the system was recording *NT* samples, but at a certain point began to register *TO* samples.

Sensitivity here is the ability of the system to classify a sample as *NT* correctly, while specificity is the aptitude of correctly classifying a sample as *TO*. A false positive in this scenario represents that the system classified an arriving sample as *TO* being *NT*, while a false negative represents that an arriving sample was classified as *NT* being *TO*. Likewise, we wanted the system to return high values of specificity and a low percentage of FPR.

The proposed system based the detection of *TO* on the existence of a non-linearity peak above 2σ, as described in Section 5. The reasons we opted for this solution were:

- A peak above 3σ was detected within the *TO* section of all subjects (specificity = 100%), indicating a clear pattern of sudden non-linearity increase in the neuronal signal of the subthalamic nucleus, just before the patient experienced physical tremor. This peak can be used as a trigger for the decision to begin stimulation by the system. It is a simple and effective system.
  Notice that despite detecting a peak above 3σ in all recordings, a conservative threshold was set at 2σ (statistical significance of the peak *p*< 0.05) in order to ensure that the peak triggered the start of stimulation in unseen futures cases, which might perhaps present a less significant peak.
- An SVM system was trained to distinguish *NT* samples from *TO*, obtaining worse results than in the previous usage scenario. This was the expected outcome since the classes to be classified were more similar between them. Remember in this regard that *TO* is a transition state between *NT* and *TO*. Results are presented in Table 2. As can be seen, the specificity did not reach 100% in any of the patients, obtaining higher values of FPR the previous use case. With the addition that in this case, the importance of correctly classifying a sample was more critical than in the previous usage case. If the window being evaluated was incorrectly classified as *NT*, being a *TO* sample, the system would continue in standby, not starting the stimulation. As soon as the patient left the *TO* state and entered the *T* state, he or she would begin to tremble (as represented in Figure 3a1). It is crucial that the system does not leave the patient needing it without stimulation. This is a red line for the system.

## 7. Related Work

Closed-loop neurostimulation is an umbrella term that encompasses different advanced DBS strategies that apply various approaches to treat the symptoms. We can find the following families of closed-loop DBS strategies [35]:

- Adaptive DBS: These methods propose a real-time adaptation of HFS parameters (the frequency, duration and amplitude of a square-wave pulse train), which are currently determined by a clinician during the visit of the patient to the hospital every 3–12 months.
- Demand-driven DBS: These strategies are based on detecting the fingerprints of pathological states and triggering the HFS as a result.
  In our opinion, the combination of adaptive and demand-driven DBS approximations would provide a complete solution for an autonomous and intelligent DBS system, able to adapt the stimulation parameters by itself and also capable of start-up and shut-down by itself as required by the changing dynamics of the STN in real time.
- Delayed DBS: These strategies consist of providing stimulation in a time-delayed manner, with the added possibility of doing it in different areas using several electrodes. The objective is to concentrate a beam of out-of-phase sinusoidal signals in the target area.
- DBS based on proportional, derivative and integral feedback: These methods propose to design a stimulation signal following the LFP signal sensed in real time. This signal can be designed proportionally to the LFP activity or regarding integral or derivative LFP.
- Optimal control strategies: These techniques base the control of the stimulation policy on finding the minimum of a defined cost function. This cost function would be adjusted to the DBS objectives, such as beta-band oscillation reduction or neuronal desynchronization.

Our study presented a proposal within the demand-driven DBS strategies. Focusing on this area, we compared the results obtained with those other studies in this sub-area that provided the level of accuracy of their systems. Wu et al. [36] proposed a system using a radial basis function neural network based on particle swarm optimization trained with the STN-LFP signal. The system reached an 89.91% accuracy. In [23], the authors examined several STN-LFP characteristics of both the time and frequency domain and characteristics based on information theory. After a feature selection process, they trained a feed-forward neural network classifier, obtaining 86% accuracy. Basu et al. [37] proposed a system combining EMG and LFP signals making use of both spectral and non-linear properties of the signals, obtaining 80.2% accuracy. In [38], the authors studied the spectral characteristics of the LFP signal, classifying it in the different states of tremor employing hidden Markov models. They obtained an accuracy level of 84%.

Finally, in two of our previous studies, we obtained a global accuracy level of 85.95% [15] and 89.5% [14], respectively. In the first one, we proposed a fuzzy inference system by using subthalamic-muscular synchronization features, whereas in the second, we designed a combined system that firstly classified the type of resting tremor presented by the patient and then trained a multi-layer perceptron with spectral features of the LFP-STN signal.

Systems that make use of both the muscle and the subthalamic signals have the disadvantage of needing an external device that senses the EMG signal for its operation. In this sense, systems that make use only of the STN-LFP signal are more functional, as they could be included in the existing DBS montage. In our opinion, this is an important characteristic to take into account.

## 8. Discussion

### 8.1. Preferred Network Measures

As discussed in Section 5, the three network measures studied showed similar behaviour. However, our results showed that the clustering measure detected more abrupt changes in the non-linearity level of the subthalamic signal, which translated into more marked peaks. Thus, we would implement the trigger for starting up the DBS based on the clustering peaks.

### 8.2. Setting of the System Parameters

Since the actual mechanism of action of DBS is still unknown, there is not a standardised process to fix the stimulation parameters (rate, pulse width and voltage), which have to be fixed by the medical staff at the time of its implantation. The clinicians can later change these parameters during patient visits to the hospital to maximise the clinical improvement of the symptoms.

In order to use the proposed closed-loop DBS system, it will be necessary to calculate the parameters τ and *m* for each subject. There are two possibilities: (1) They can be fixed, as with the remainder of the parameters, at the time of implantation and reconsidered during the subsequent visits to the doctor or (2) they can be calculated from each window data in real time.

This decision will have to be made in the design phase of the IMD. The second option is more accurate, in so far as the value of the parameters is data-driven and performed in real time, and also entails a higher computation. This is inconvenient because neurostimulators, as with any other IMD, have restricted capabilities of energy, storage and computing power [39].

Considering these restrictions, we would lean towards the first option. In this case, we need to test the robustness of the proposed method against the parameters τ and *m*. It is necessary to know to what extent these parameters affect the detection of the tremor states and transitively the detection of the tremor, i.e., the robustness of the proposed approach.

**Figure 5:**
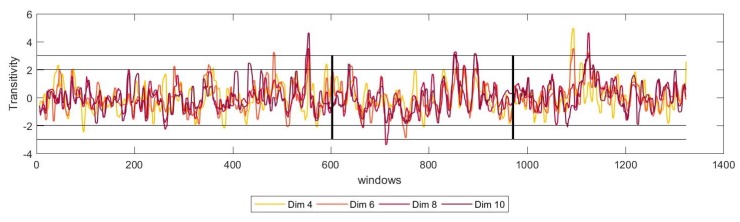
Moving window ε-recurrence network analysis showing the median moving average of transitivity, before, during and after the start of the tremor. The temporal profile of the measure is shown for different values of m=4,6,8 and 10. The vertical line represent the time at which the patients transited from *NT* to *TO* (left) and from *TO* to *T* (right). The horizontal lines represent the ±2 and ±3 standard deviations, the thresholds for statistical significance.

For that matter, we have studied the dynamics of the system for several numbers of dimensions, *m*, of the reconstructed phase space. The results showed that for all the possible number of dimensions, the network measured show a similar trend, stabilising for higher values of *m*. Figure 5 shows these results for transitivity for one of the patients.

From these results, we can conclude that, even if the number of dimensions necessary to reconstruct the phase state change slightly, this would not affect the performance in the detection of the tremor. Nevertheless, more studies in this direction would be necessary.

For its part, the value of τ across windows has a very low variance, oscillating in a unit. For example, given a patient for which the optimal value of τ for most of the windows is τ=4, we could find some windows of this same patient with an optimal value of τ=3. In this case, we would set τ=4.

Nevertheless, we have found that the performance of the system was suitable across all the windows, and there was not an appreciable effect on the prediction of the peak before the onset of the tremor. Hence, we can conclude that the system was robust against the choice of the parameter τ, at least in our case, in which this parameter had a minimal variance.

### 8.3. Towards Future DBS Systems

DBS has proven to be an effective solution for the treatment of movement disorders, especially in cases where oral treatment is not enough. However, continued stimulation may induce adverse effects, while the device’s battery is not used efficiently. These two drawbacks of the DBS can be mitigated using new closed-loop strategies. In the case of demand-driven DBS strategies, the objective is to adapt the functioning of the device in real time in response to changes in clinical (motor) status experienced by the patient. Several studies, including significant device development companies, agree that closed-loop strategies will be the therapy implemented in future DBS systems. To this end, the new generation of DBS devices must be able to sense the electrical signal in the target area while simultaneously delivering therapy. These devices will be able to obtain artefact-free LFP recordings during stimulation. For this purpose, from the industry side, Medtronic developed the *Activa^®^ PC+S* neurostimulator, which is only available for research so far, but that points the way to future neurostimulation systems [40].

## 9. Conclusions and Future Work

The behaviour of the STN becomes highly non-linear during tremor episodes, when compared with the basal state (*NT*), making the geometry of the phase state more structured. We hypothesize that the witnessed increase in nonlinearity, as reflected by the shift in the network measures, could be attributed to the change in synchronization between the neurons during the tremor episode, as seen in a previous study [19].

ε-recurrence network analysis is a suitable method to distinguish the transitions between movement conditions. Furthermore, the implemented method has the advantage of being able to deal with both short and non-stationary data, making it a good option for LFP data. These two facts make this procedure appropriate for its application to a closed-loop DBS system.

The setting of the parameters of the system, τ and *m*, can be taken at the time of the start-up of the device and adapted if necessary, during the visits of the patient to the clinician. This solution takes into consideration the inherent constraints of the IMD: energy, storage and computing power, making viable the implementation of the proposed solution.

Given the difficulty of getting STN recordings like those used in this work, only four patients have been studied. Despite having found very similar results in all of them, it would be necessary to consolidate these results in more patients. Our intention with this work is to propose that ε-recurrence networks may be a useful tool in the design of systems that interact with brain signals, not only in PD [6,22], since all neural activity is a source of non-linear, non-stationary data.

## Figures and Tables

**Table 1:** SVM-subsystem performance in stopping the stimulation.

File	ACC	Sensitivity	Specificity	FPR	FNR
1	84.8	89.83	79.24	20.75	10.17
2	98.4	97.44	100	0	2.56
3	94.3	95.20	90.8	9.2	4.79
4	90.3	87.13	92.05	7.94	12.86

**Table 2:** SVM-subsystem performance in starting the stimulation.

File	ACC	Sensitivity	Specificity	FPR	FNR
1	69.6	74.54	50	50	25.45
2	77.1	60	81.57	18.42	40
3	86.7	80	92.06	7.93	20
4	82.1	81.39	84	16	18.6

## References

[1] Dorsey E.R., Bloem B.R. (2018). The parkinson pandemic–A call to action. JAMA Neurol..

[2] Farrer M.J. (2006). Genetics of parkinson disease: paradigm shifts and future prospects. Nat. Rev. Genet..

[3] Josephs K.A., Matsumoto J.Y., Ahlskog J.E. (2006). Benign tremulous parkinsonism. Arch. Neurol..

[4] da Silva F.H.L., Blanes W., Kalitzin S.N., Parra J., Suffczynski P., Velis D.N. (2003). Dynamical diseases of brain systems: Different routes to epileptic seizures. IEEE Trans. Biomed. Eng..

[5] Mackey M.C., Milton J.G. (1987). Dynamical diseases. Ann. N. Y. Acad. Sci..

[6] Subramaniyam N.P., Donges J.F., Hyttinen J. (2015). Signatures of chaotic and stochastic dynamics uncovered with *ε*-recurrence networks. Proc. R. Soc. A.

[7] Nof S.Y. (2009). Springer Handbook of Automation.

[8] Bezard E., Brotchie J.M., Gross C.E. (2001). Pathophysiology of levodopa-induced dyskinesia: Potential for new therapies. Nat. Rev. Neurosci..

[9] Fabbrini G., Brotchie J.M., Grandas F., Nomoto M., Goetz C.G. (2007). Levodopa-induced dyskinesias. Mov. Disord. Off. J. Mov. Disord. Soc..

[10] Benabid A.L., Chabardes S., Mitrofanis J., Pollak P. (2009). Deep brain stimulation of the subthalamic nucleus for the treatment of parkinson’s disease. Lancet Neurol..

[11] Sugiyama K. (2014). Complications of Deep Brain Stimulation.

[12] Priori A., Foffani G., Rossi L., Marceglia S. (2013). Adaptive deep brain stimulation (adbs) controlled by local field potential oscillations. Exp. Neurol..

[13] Little S., Beudel M., Zrinzo L., Foltynie T., Limousin P., Hariz M., Neal S., Cheeran B., Cagnan H., Gratwicke J. (2015). Bilateral adaptive deep brain stimulation is effective in parkinson’s disease. J. Neurol. Neurosurg. Psychiatry.

[14] Camara C., Isasi P., Warwick K., Ruiz V., Aziz T., Stein J., Bakštein E. (2015). Resting tremor classification and detection in parkinson’s disease patients. Biomed. Signal Proc. Control.

[15] Camara C., Warwick K., Bruña R., Aziz T., del Pozo F., Maestú F. (2015). A fuzzy inference system for closed-loop deep brain stimulation in parkinson’s disease. J. Med. Syst..

[16] Kühn A.A., Kupsch A., Schneider G., Brown P. (2006). Reduction in subthalamic 8–35 Hz oscillatory activity correlates with clinical improvement in parkinson’s disease. Eur. J. Neurosci..

[17] Weinberger M., Hutchison W.D., Dostrovsky J.O. (2009). Pathological subthalamic nucleus oscillations in PD: Can they be the cause of bradykinesia and akinesia?. Exp. Neurol..

[18] Hohlefeld F.U., Huchzermeyer C., Huebl J., Schneider G., Nolte G., Brücke C., Schönecker T., Kühn A.A., Curio G., Nikulin V.V. (2013). Functional and effective connectivity in subthalamic local field potential recordings of patients with parkinson’s disease. Neuroscience.

[19] Camara C., Warwick K., Bruña R., Aziz T., Pereda E. (2019). Closed-loop deep brain stimulation based on a stream-clustering system. Expert Syst. Appl..

[20] da Silva F.L., Blanes W., Kalitzin S.N., Parra J., Suffczynski P., Velis D.N. (2003). Epilepsies as dynamical diseases of brain systems: Basic models of the transition between normal and epileptic activity. Epilepsia.

[21] Donner R.V., Zou Y., Donges J.F., Marwan N., Kurths J. (2010). Recurrence networks–A novel paradigm for nonlinear time series analysis. N. J. Phys..

[22] Subramaniyam N.P., Hyttinen J., Hatsopoulos N.G., Takahashi K. Recurrence network analysis of wide band oscillations of local field potentials from the primary motor cortex reveals rich dynamics. Proceedings of the 7th International IEEE/EMBS Conference on Neural Engineering (NER).

[23] Bakstein E., Burgess J., Warwick K., Ruiz V., Aziz T., Stein J. (2012). Parkinsonian tremor identification with multiple local field potential feature classification. J. Neurosci. Methods.

[24] Stam C.J. (2005). Nonlinear dynamical analysis of eeg and meg: review of an emerging field. Clin. Neurophysiol..

[25] Takens F. (1981). Detecting strange attractors in turbulence. Dynamical Systems and Turbulence, Warwick 1980.

[26] Fraser A.M., Swinney H.L. (1986). Independent coordinates for strange attractors from mutual information. Phys. Rev. A.

[27] Kantz H., Schreiber T. (2004). Nonlinear Time Series Analysis.

[28] Kennel M.B., Brown R., Abarbanel H.D.I. (1992). Determining embedding dimension for phase-space reconstruction using a geometrical construction. Phys. Rev. A.

[29] Donner R.V., Small M., Donges J.F., Marwan N., Zou Y., Xiang R., Kurths J. (2011). Recurrence-based time series analysis by means of complex network methods. Int. J. Bifurc. Chaos.

[30] Marwan N., Donges J.F., Zou Y., Donner R.V., Kurths J. (2009). Complex network approach for recurrence analysis of time series. Phys. Lett. A.

[31] Small Michael (2005). Applied Nonlinear Time Series Analysis: Applications in Physics, Physiology and Finance.

[32] Schank T., Wagner D. (2004). Approximating Clustering-Coefficient and Transitivity.

[33] Newman M.E.J. (2002). Assortative mixing in networks. Phys. Rev. Lett..

[34] Cristianini N., Shawe-Taylor J. (2000). An introduction To Support Vector Machines and Other Kernel-Based Learning Methods.

[35] Carron R., Chaillet A., Filipchuk A., Pasillas-Lépine W., Hammond C. (2013). Closing the loop of deep brain stimulation. Front. Syst. Neurosci..

[36] Wu D., Warwick K., Ma Z., Gasson M.N., Burgess J.G., Pan S., Aziz T.Z. (2010). Prediction of parkinson’s disease tremor onset using a radial basis function neural network based on particle swarm optimization. Int. J. Neural Syst..

[37] Basu I., Graupe D., Tuninetti D., Shukla P., Slavin K.V., Metman L.V., Corcos D.M. (2013). Pathological tremor prediction using surface electromyogram and acceleration: Potential use in ‘on–off’demand driven deep brain stimulator design. J. Neural Eng..

[38] Hirschmann J., Schoffelen J.M., Schnitzler A., van Gerven M.A.J. (2017). Parkinsonian rest tremor can be detected accurately based on neuronal oscillations recorded from the subthalamic nucleus. Clin. Neurophysiol..

[39] Camara C., Peris-Lopez P., Tapiador J.E. (2015). Security and privacy issues in implantable medical devices: A comprehensive survey. J. Biomed. Inf..

[40] Medtronic Activa PC+S Deep Brain Neurostimulator. https://medtronicmediacap.gcs-web.com/new-medtronic-deep-brain-stimulation-system-first-sense-and-record-brain-activity-while.

